# Analytical investigation of an incompressible viscous laminar Casson fluid flow past a stretching/shrinking sheet

**DOI:** 10.1038/s41598-022-23295-6

**Published:** 2022-11-01

**Authors:** Ulavathi Shettar Mahabaleshwar, Thippaiah Maranna, Filippos Sofos

**Affiliations:** 1 Department of Studies in Mathematics, Davangere University, Shivagangothri, Davangere, 577 007 India; 2 Condensed Matter Physics Laboratory, Department of Physics, University of Thessaly, 35100 Lamia, Greece

**Keywords:** Nanoparticles, Applied mathematics

## Abstract

This paper presents an analytical approach on capturing the effect of incompressible, non-Newtonian, viscous, Casson nanofluid flow past a stretching/shrinking surface, under the influence of heat radiation and mass transfer parameter. The governing nonlinear partial differential equations are first transformed into a series of associated nonlinear ordinary differential equations with aid of predictable transformation, while numerical computations follow. The implied nanofluid here is aluminum oxide ($Al_{2} O_{3}$). The analytical solution is exploited to reveal the accompanying non-dimensional boundary value problem and output results are employed to verify the method's reliability, where it is shown that they agree with current findings in the field. An incomplete gamma function is used to solve temperature equation analytically. We present various instances of the solution, depicting effects of the essential flow factor, the stretching/shrinking parameter, the mass transfer parameter, radiation parameter, and Prandtl number.

## Introduction

Non-Newtonian fluids have been exploited in numerous applications nowadays, such as the flow of nuclear fuel slurries, liquid metal and alloy flows, plasma flows, mercury amalgam flows, and flows for lubrication with heavy oil and greases, coating of papers, polymer extrusion, and numerous other processes. It is of particular importance that, understanding non-Newtonian fluid dynamics, whether with or without heat transfer, can lead to better acquiring of concepts like food freezing and polymer injection, and others. A broad range of fluids, such as salt solutions, melted sauce, custard, toothpaste, starch syrup, paints, blood, or shampoo, exhibit remarkable properties due to highly viscous behavior. The fundamental theory of stress and strain velocity that applies well in Newtonian fluids cannot be applied in such cases, and the term non-Newtonian has long been incorporated^1^. Benefits that stem from the exploitation of non-Newtonian fluids, apart from drag reduction^2^, are their advanced thermal properties.

During the last decades, a lot of research has been made on the nanofluid flow and heat transfer with water as a base fluid. Among them, nanofluids, as introduced by Choi et al.^3^, have been recommended in suspending nanoparticles in a base fluid such as water, oil, or ethylene glycol. Tiwari et al.^4^, have proposed various nanofluidic concepts, which can aid in understanding convective recirculation and flow processes induced by a nanofluid. Hwang et al.^5^ have investigated nanofluids in terms of thermal conductivity and come to the conclusion that the volume fraction of nanoparticles, its characteristics, and the base fluids’ thermal properties are significant factors that affect thermal conductivity. Arash et al.^6^ have implemented a model of a nanofluid inside a microchannel under the effect of a magnetohydrodynamic field. By considering a *Cu–H*_*2*_*O* nanofluid, it has been also found that Cu nanoparticles lead to higher nanofluid temperature upon heating^7^.

Further studies include nanofluid research across a stretched/shrinking sheet with an impact of radiation and mass transpiration^8–11^. By adopting a dual transpiration approach, a precise method for entropy production in a magnetohydrodynamic flow of nanofluid caused by stretched/shrinking surface was developed by Freidoonimehr et al.^12^. In tunnels with turbulent flow, Xuan et al.^13^, have measured properties relevant to the nanoparticle flow and heat transfer. As stated by their experimental findings, increasing the volume fraction, as well as the Reynolds number of nanostructures, can improve heat transfer by convection and the Nusselt number of nanofluids. An exponential stretched surface affected by a magnetic field, chemical processes, heat flux, as well as viscous dissipation, produce steady movement of the boundary layer of nanofluid, and mathematical formulation of this problem is proposed by Reddy et al.^14^.

Recently, Sneha et al*.*^15^ have investigated carbon nanotubes (CNTs) characteristics, in terms of their water-based nanoparticles and dusty hybrid nanofluid flow for the Darcy-Brinkman model under conditions of radiation and mass transpiration. Anusha et al.^16^ have studied how the application of MHD has affected the flow of nanofluids, dusty hybrid nanofluids at its stagnation point through permeable stretching/shrinking surfaces, under the effects of mass transpiration and heat flux. Fang et al.^17^ have analyzed the performance of stable boundary layer flow transfer of an inviscid and viscoelastic fluid approaching porous stretching/shrinking sheets. Mandal et al.^18^ have examined the impact of convective heat, viscosity dissipation generated by nanomaterials, and the induced magnetic field.

Casson fluid is classified as a non-Newtonian fluid due to its rheological characteristics in relation to the shear stress–strain relationship. It behaves like an elastic solid at low shear strain and above a critical stress value, it behaves like a Newtonian fluid. A Casson fluid can better described as a shear thinning liquid with infinite viscosity at zero shear rate, and zero viscosity at an infinite rate of shear. Some common examples of liquids that exhibit Casson fluid characteristics include tomato sauce, honey, soup, orange juice and human blood. Recently, exact solutions have been established by a novel technique for the effects of heat and mass transfer on the peristaltic flow of non-Newtonian Casson fluid inside an elliptic conduit, studied by Akhtar et al.^19^ The physiological stream of Casson fluid in a vertical elliptical duct with heated, ciliated surfaces has been estimated analytically by Fuzhang et al.^20^.

On the other hand, several studies have been conducted to analyze the Magnetohydrodynamic Casson fluid, such as Casson nanofluid flow over a nonlinear slanted extending/shrinking surface, oscillating disk in Darcy–Forchheimer medium under the effect of heat and mass transfer, thermal energy in terms of heat source/sink, thermal radiation and chemical reaction, while a numerical analysis has been conducted for the three-dimensional flow of a hybrid nanofluid under/over a stretching surface using supervised Neural Networks^21–24^. More recently, the injection of water-based nanoparticle (NP) suspensions has received attention as a recovery enhancement technique. Awais et al.^25^ has theoretically studied the influence of Hall and slip with temperature-dependent viscosity, by conceptually exposing the rheological behavior of copper water nanofluid peristaltic flow through generalized flexible surfaces. Furthermore, recent studies have reported nanofluidic effects and enhanced heat transmission in dispersion of micropolar fluids utilizing the KKL model, entropy generation and rate of heat transfer in steady flow of (*Al*_*2*_*O*_*3*_*–Cu/H*_*2*_*O*) hybrid nanofluid due to radially stretching disk by imposing convective-type thermal conditions, and the three-dimensional Oldroyd’s-B fluid with nonlinear thermal radiation past over the stretched surface (see Awais et al.^26–28^).

The present article aims at investigating the following aspects:

- the steady laminar boundary layer flow and heat transfer of a viscous and an incompressible non-Newtonian fluid over a linearly stretching/shrinking surface.
- Casson fluid model is utilized to describe the non-Newtonian fluid behavior. This type of fluid has wide applications in food processing, in metallurgy, drilling operation and bio-engineering operations.
- The nanofluid, that is, *Al*_*2*_*O*_*3*_-water is studied.
- The thermal radiation effect in such configuration is also studied.
- Dimensionless expressions of velocity and temperature are solved analytically.
- The presented plots illustrate the behavior of pertinent parameters such as stretching/shrinking parameter, mass transpiration, Prandtl number, radiation and free parameter on the velocity and temperature.
- The novelty of this work is to evaluate the effect of thermal radiation on laminar boundary layer flow of Casson nanofluid through a stretching/shrinking sheet. The physical quantities like skin friction and Nusselt number are also evaluated. Finally, we hope that the results of this study will be applicable to processes like extrusion, cord depiction, copper spiraling, heat progressing, and melts of high molecular weight polymers.

## Methods

### Model and mathematical formulation

**Figure 1:**
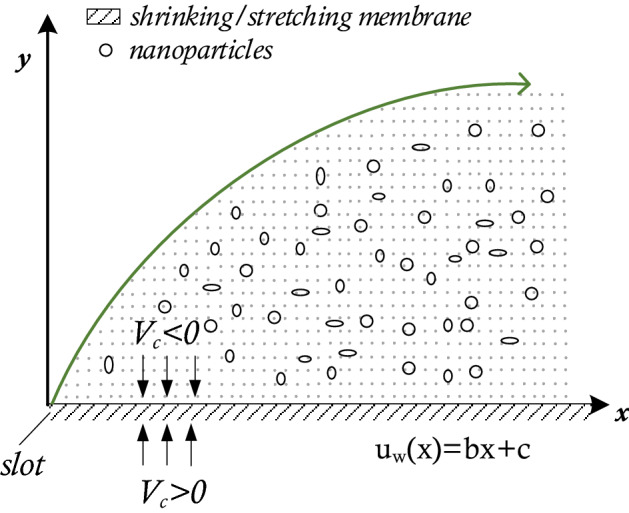
Schematic representation of the implied problem.

We consider the steady, two-dimensional non-Newtonian flow of an incompressible viscous fluid past a stretching/shrinking sheet with mass transfer in a stationary fluid, as shown in Fig. 1. Here, the stretching surface moving velocity is given by $u_{w} \left( x \right) = bx + C,$ where the stretching rate is for $b > 0,$ and shrinking rate for $b < 0$, and *C* is the constant velocity component. There is a constant mass transfer velocity at the wall is $V_{w}$ together with $V_{w} > 0$ for suction, while, for injection we have $V_{w} < 0$. At $T_{w}$, surface temperature is constant with fixed temperature of the ambient fluid at $T_{\infty }$. The *x*-axis is measured along the stretching surface and *y*-axis is perpendicular to it.

The rheological model for the flow of a Casson fluid can be written as

1 $$\tau_{ij} \, = \,\left\{ \begin{gathered} \,2\left( {\mu_{B} + \frac{{p_{y} }}{{\sqrt {2\pi } }}} \right)e_{ij} ,\quad \pi > \pi_{c} ,\, \hfill \\ \,2\left( {\mu_{B} + \frac{{p_{y} }}{{\sqrt {2\pi } }}} \right)e_{ij} ,\quad \pi < \pi_{c} . \hfill \\ \end{gathered} \right.$$

Here $\mu_{B}$ is plastic dynamics viscosity of the Casson fluid, $p_{y}$ is the yield stress of fluid, the product of the component of resultant deformation rate with itself is $\pi$. Namely, $\pi = e_{ij} e_{ij}$, $e_{ij}$ is the $\left( {i,j} \right)$th components of the deformation rate, and product's critical value is denoted as $\pi_{c}$.

The basic two-dimensional boundary layer momentum and energy equations are defined as^29–32^

2 $$\frac{\partial u}{{\partial x}} + \frac{\partial v}{{\partial y}} = \,0,$$

3 $$u\frac{\partial u}{{\partial x}} + v\frac{\partial u}{{\partial y}}\, = \, - \frac{1}{{\rho_{nf} }}\frac{\partial p}{{\partial x}} + v_{nf} \left( {1 + \frac{1}{\Lambda }} \right)\frac{{\partial^{2} u}}{{\partial y^{2} }},$$

4 $$u\frac{\partial u}{{\partial x}} + v\frac{\partial v}{{\partial y}} = - \frac{1}{{\rho_{nf} }}\frac{\partial p}{{\partial y}} + v_{nf} \left( {1 + \frac{1}{\Lambda }} \right)\frac{{\partial^{2} v}}{{\partial y^{2} }},$$

5 $$u\frac{\partial T}{{\partial x}} + v\frac{\partial T}{{\partial y}}\, = \,\chi \frac{{\partial^{2} T}}{{\partial y^{2} }} - \frac{1}{{\left( {\rho C_{p} } \right)_{nf} }}\frac{{\partial q_{r} }}{\partial y},$$

where $u$ and $v$ are the $x$- and $y$-components of velocity, respectively, $p$: pressure, $\rho_{nf}$: nanofluid density, $v_{nf}$: nanofluid kinematic viscosity, $\chi = \frac{{\kappa_{nf} }}{{\left( {\rho C_{p} } \right)_{nf} }}$: nanofluid thermal diffusivity, with $\kappa_{nf}$ the nanofluid thermal conductivity, fluid temperature is $T$, $\left( {\rho C_{p} } \right)_{nf}$ is nanofluid heat capacitance and, finally, the Casson parameter is $\Lambda$.

The unsteady basic governing equations’ boundary constraints are

6 $$\left. {\begin{array}{*{20}c} {u = u_{w} \left( x \right) = bx + C,} & {v = v_{w} ,} & {T = T_{w} } & {{\text{at}}\quad y = 0,} \\ {u = 0,} & {T = T_{\infty } ,} & {} & {{\text{as}}\quad {\text{y}} \to \infty } \\ \end{array} } \right\},$$

### Similarity variables

The advantages of the similarity transformation are to convert the Partial Differential Equations into highly non-linear Ordinary Differential Equations. In order to simplify the analysis of the problem, we proposed the correct self-similarity variables mentioned below^17,29^

7 $$\left. \begin{array}{lll} u\, = \,ax\frac{\partial f}{{\partial \eta }} + CF\left( \eta \right), & v\, = \, - \sqrt {av_{f} } f\left( \eta \right),&\,\,\eta \, = \,\sqrt {\frac{a}{{v_{f} }}} y, \\ T - T_{\infty } \, = \,\theta \left( \eta \right)\left( {T_{w} - T_{\infty } } \right), & & \end{array} \right\},$$

where $a$ is constant (always positive for this condition), the reference velocity is represented as $u_{r} \left( x \right) = ax$, and $u = u_{r} \left( x \right)\frac{\partial f}{{\partial \eta }} + Cg\left( \eta \right)$. We present the following expression for the pressure $p$ using Eq. (4) and the boundary conditions (6),

8 $$p\, = \,p_{0} - \rho \frac{{v^{2} }}{2} + \rho v\frac{\partial v}{{\partial y}},$$

where stagnation pressure is $p_{0}$.

By using Rosseland’s diffusion approximation for radiation and following methodology shown in^33–36^, the radiative flux $q_{r}$ is of the form

9 $$q_{r} = - \frac{{4\sigma^{*} }}{{3\kappa^{*} }}\frac{{\partial T^{4} }}{\partial y},$$

where $\sigma^{*}$ and $\kappa^{*}$ are Stefan–Boltzmann values and the mean absorption factor. It is assumed that temperature variations within the flow are minimal enough to interpret the radiative flux’s fourth-power term as a linear function of temperature, as

10 $$T^{4} \cong \,4T_{\infty }^{3} T - 3T_{\infty }^{4} ,$$

Now, by differentiating Eq. (9) with respect to *y*, we get

11 $$\frac{{\partial q_{r} }}{\partial y}\, = \, - \frac{{16\sigma^{*} T_{\infty }^{3} }}{{3k^{*} }}\frac{{\partial^{2} T}}{{\partial y^{2} }},$$

and by substituting Eq. (7) into Eq. (3), (4), and (5), we have following ordinary differential equations,

12 $$A_{1} \left( {1 + \frac{1}{\Lambda }} \right)\frac{{\partial^{3} f}}{{\partial \eta^{3} }} + A_{2} \left( {f\left( \eta \right)\frac{{\partial^{2} f}}{{\partial \eta^{2} }} - \left( {\frac{\partial f}{{\partial \eta }}} \right)^{2} } \right)\, = \,0,\,$$

13 $$A_{1} \left( {1 + \frac{1}{\Lambda }} \right)\frac{{\partial^{2} F}}{{\partial \eta^{2} }} + A_{2} \left( {f\left( \eta \right)\frac{\partial F}{{\partial \eta }} - \frac{\partial f}{{\partial \eta }}F\left( \eta \right)} \right)\, = \,0,$$

14 $$A_{3} \left( {1 + N_{r} } \right)\frac{{\partial^{2} \theta }}{{\partial \eta^{2} }} + \Pr A_{4} \left( {f\left( \eta \right)\frac{\partial \theta }{{\partial \eta }}} \right)\, = \,0,$$

where

15 $$A_{1} = \frac{{\mu_{nf} }}{{\mu_{f} }},\quad A_{2} = \frac{{\rho_{nf} }}{{\rho_{f} }},\quad A_{3} = \frac{{\kappa_{nf} }}{{\kappa_{f} }},\quad {\text{and}}\quad A_{4} = \frac{{\left( {\rho C_{p} } \right)_{nf} }}{{\left( {\rho C_{p} } \right)_{f} }},$$

and Prandtl number is $\Pr = \frac{{v_{f} \left( {\rho C_{p} } \right)_{f} }}{{k_{f} }},$ while the expression for this nanofluid can be denoted as^37–40^.

16 $$\begin{aligned} \mu_{nf} \, &= \,\frac{{\mu_{f} }}{{\left( {1 - \varphi } \right)^{2.5} }}, \\ \rho_{nf} \, &= \,\left( {\left( {1 - \varphi } \right) + \varphi \left( {\frac{{\rho_{s} }}{{\rho_{f} }}} \right)} \right)\rho_{f} , \\ \kappa_{nf} &\, = \,\frac{{\kappa_{f} \left( {\left( {\kappa_{s} + 2\kappa_{f} } \right) - 2\varphi \left( {\kappa_{f} - \kappa_{s} } \right)} \right)}}{{\left( {\left( {\kappa_{s} + 2\kappa_{f} } \right) + \varphi \left( {\kappa_{f} - \kappa_{s} } \right)} \right)}},\,\,\,\,\,\, \\ \left( {\rho C_{p} } \right)_{nf} \, &= \,\left( {\rho C_{p} } \right)_{f} \left( {\left( {1 - \varphi } \right) + \varphi \left( {\frac{{\left( {\rho C_{p} } \right)_{s} }}{{\left( {\rho C_{p} } \right)_{f} }}} \right)} \right), \hfill \\ \end{aligned}$$

where the base fluid dynamic viscosity is $\mu_{f}$, nanofluid dynamic viscosity is $\mu_{nf}$, thermal conductivity of the base fluid is $\kappa_{f}$, heat capacitance of fluid is $\left( {\rho C_{p} } \right)_{f}$, nanofluid volume fraction is $\varphi$.

The transformed boundary conditions are

17 $$\left. {\begin{array}{*{20}c} {f\left( \eta \right)_{\eta \, = \,0} = V_{c} ,} & {\left( {\frac{\partial f}{{\partial \eta }}} \right)_{\eta \, = \,0} = \frac{b}{a\,} = d,} & {F\left( \eta \right)_{\eta \, = \,0} = 1,} \\ {\left( {\frac{\partial f}{{\partial \eta }}} \right)_{\eta \to \infty } = 0,} & {F\left( \eta \right)_{\eta \to \infty } = 0,} & {\theta \left( \eta \right)_{\eta \to \infty } = 0,} \\ \end{array} } \right\},$$

where $V_{c}$ is the mass transpiration constant, with suction case $V_{c} > 0$ and $V_{c} < 0$ for case of injection, $d$ is the stretching/shrinking factor, giving $d > 0$ for stretching and $d < 0$ for shrinking.

### Thermophysical properties

The experimental values of $Cp$ (specific heat), $\rho$ (density), and $\kappa$ (thermal conductivity), for the base fluid (water), and nanofluid $\left( {Al_{2} O_{3} } \right)$ are given in Table 1, according to^41–43^.

**Table 1:** Thermophysical properties of fluids and nanofluids studied here.

Nanoparticles/base fluid	$\rho$ (kg m^−3^)	*k* (W m^−1^ K^−1^)	*C*_*p*_ (J kg^−1^ K^−1^)
Water (*H*_2_*O*)	997.8	0.604	4076.4
Aluminum oxide (*Al*_2_*O*_3_)	3970	40	765

By using non-dimensional transformation (Eq. 7), the physical quantity of interest is the dimensionless skin friction factor is defined as

18 $$C_{f} = \,\frac{{\tau_{w} }}{{\rho u_{r}^{2} }},$$

where $\tau_{w}$ is the skin friction or shear stress and is described as

19 $$\tau_{w} \, = \,\mu \left( {\frac{\partial u}{{\partial y}}} \right)_{y = 0} ,$$

and when we use the similarity variable, we get the local skin friction coefficient as follows

20 $${\text{Re}}_{x}^{\frac{1}{2}} C_{f} \, = \,\left( {\frac{{\partial^{2} f}}{{\partial \eta^{2} }}} \right)_{\eta \, = \,0} + \frac{C}{{u_{r} }}\left( {\frac{\partial F}{{\partial \eta }}} \right)_{\eta \, = \,0} ,$$

where ${\text{Re}}_{x} = \frac{{u_{r} x}}{v}$ is the local Reynolds number.

Streamlines normalized for this flow are defined as

21 $$\overline{\psi } \, = \,xf\left( \eta \right) + \frac{C}{a}\int_{0}^{\eta } {F\left( s \right)} ds,$$

where $\overline{\psi } = \frac{\psi }{{\left( {av} \right)^{\frac{1}{2}} }}$, with $\psi$ interpreted in the normal way as $u = \frac{\partial \psi }{{\partial y}}$ with $w = \frac{ - \partial \psi }{{\partial x}}$.

### Obtaining the velocity solution

Exact solution of the momentum equation is considered in the following form:

22 $$f\left( \eta \right)\, = \,\beta + \left( {V_{c} - \beta } \right)\exp [ - \beta \eta ]\,\, = \,\,\beta - \frac{d}{\beta }\exp [ - \beta \eta ]\,,$$

with its first order derivative is follows

23 $$\frac{\partial f}{{\partial \eta }}\, = \, - \beta \left( {V_{c} - \beta } \right)\exp [ - \beta \eta ]\, = \,d\exp [ - \beta \eta ]\,,$$

Now Eq. (12) becomes,

24 $$A_{1} \left( {1 + \frac{1}{\Lambda }} \right)\beta^{2} - A_{2} V_{c} \beta - A_{2} d\, = \,0,$$

which is a second-degree algebraic equation, and gives two pair of real roots, such as

25 $$\beta \, = \,\frac{{A_{2} V_{c} \pm \sqrt {\left( {A_{2} V} \right)^{2} + 4A_{1} A_{2} \left( {1 + \frac{1}{\Lambda }} \right)} }}{{2A_{1} \left( {1 + \frac{1}{\Lambda }} \right)}},$$

The roots can be rewritten as

$$\beta_{1} = \frac{{A_{2} V_{c} + \sqrt {\left( {A_{2} V} \right)^{2} + 4A_{1} A_{2} \left( {1 + \frac{1}{\Lambda }} \right)} }}{{2A_{1} \left( {1 + \frac{1}{\Lambda }} \right)}},\quad \beta_{2} = \frac{{A_{2} V_{c} - \sqrt {\left( {A_{2} V} \right)^{2} + 4A_{1} A_{2} \left( {1 + \frac{1}{\Lambda }} \right)} }}{{2A_{1} \left( {1 + \frac{1}{\Lambda }} \right)}},$$

Consequently, Eq. (13) takes the form

26 $$A_{1} \left( {1 + \frac{1}{\Lambda }} \right)\frac{{\partial^{2} F}}{{\partial \eta^{2} }} + A_{2} \left( {\beta + \left( {V_{c} - \beta } \right)\exp [ - \beta \eta ]} \right)\frac{\partial F}{{\partial \eta }} + \beta \left( {V_{c} - \beta } \right)\exp [ - \beta \eta ]F\left( \eta \right)\, = \,0,$$

There seems to be a novel solution for $F\left( \eta \right) = \frac{\partial f}{{\partial \eta }} = d\exp [ - \beta \eta ],$ and the complete solution to Eq. (25) is

27 $$F\left( \eta \right)\, = \,A\exp [\beta \eta ] + B\left\{ { - \exp \left( {\frac{{\left( {V_{c} - \beta } \right)\exp [ - \beta \eta ]}}{\beta }} \right) + \frac{{\exp [ - \beta \eta ]E_{i} \left( {\frac{{\left( {V_{c} - \beta } \right)\exp [\beta \eta ]}}{\beta }} \right)}}{\beta }} \right\},$$

in which $Ei\left( x \right) = - \mathop \smallint_{ - x}^{\infty } \left( {\frac{\exp [ - t]}{t}} \right)dt$ is a function of exponential integral, since $A$ and $B$ are two constants of integration. When $\eta \to \infty ,\;F\left( \eta \right) \to 0$. $B = 0$ and $A = 1$ are found. Hence, the equation to $F\left( \eta \right)$ is

28 $$F\left( \eta \right)\, = \,\exp [ - \beta \eta ],$$

Consequently, the velocity components appear as follow

29 $$u\, = \,bx\exp [ - \beta \eta ] + C\exp [ - \beta \eta ],$$

with

30 $$v\, = \, - \sqrt {av} \left( {\beta + \left( {V_{c} - \beta } \right)\exp [ - \beta \eta ]} \right),$$

Non-dimensional stream function is now transformed into modified form given by

31 $$\widetilde{\psi }\, = \,xf\left( \eta \right) + \frac{C}{a}\int_{0}^{\eta } {F\left( {V_{c} } \right)} dV_{c} \, = \,\beta x + x\left( {V_{c} - \beta } \right)\exp [ - \beta \eta ] - \frac{C}{a\beta }\exp [ - \beta \eta ],$$

Next, an algebraically decaying solution follows, produced from the momentum Eq. (12) and the boundary conditions from Eq. (17).

32 $$f\left( \eta \right)\, = \,\frac{{6A_{1} \left( {1 + \frac{1}{\Lambda }} \right)}}{{\eta A_{2} + A_{1} \sqrt {\frac{{ - 6\left( {1 + \frac{1}{\Lambda }} \right)}}{{A_{1} d}}} }},$$

in association with

33 $$\frac{\partial f}{{\partial \eta }}\, = \,\frac{{ - 6A_{1} \left( {1 + \frac{1}{\Lambda }} \right)}}{{A_{2} \left( {\eta A_{2} + A_{1} \sqrt {\frac{{ - 6\left( {1 + \frac{1}{\Lambda }} \right)}}{{A_{1} d}}} } \right)^{2} }},$$

It becomes clear that only shrinking sheets (as a consequence of $d < 0$) are impacted by the algebraically decaying function, therefore mass suction at the surface is

34 $$f\left( \eta \right)_{\eta = 0} \, = \,V_{c} \, = \,\sqrt { - 6\left( {1 + \frac{1}{\Lambda }} \right)dA_{1} } ,$$

We rewrite Eq. (13) as

35 $$A_{1} \left( {1 + \frac{1}{\Lambda }} \right)\frac{{\partial^{2} F}}{{\partial \eta^{2} }}\, + \left( {\frac{{6A_{1} \left( {1 + \frac{1}{\Lambda }} \right)}}{{\eta A_{2} + A_{1} \sqrt {\frac{{ - 6\left( {1 + \frac{1}{\Lambda }} \right)}}{{A_{1} d}}} }}} \right)\frac{\partial F}{{\partial \eta }} - \left( {\frac{{ - A_{1} 6\left( {1 + \frac{1}{\Lambda }} \right)}}{{A_{2} \left( {\eta A_{2} + A_{1} \sqrt {\frac{{ - 6\left( {1 + \frac{1}{\Lambda }} \right)}}{{A_{1} d}}} } \right)^{2} }}} \right)F\left( \eta \right)\, = \,0,$$

The complete general solution for Eq. (35) is

36 $$F\left( \eta \right)\, = \,\frac{{C_{1} }}{{\left( {\eta A_{2} + A_{1} \sqrt {\frac{{ - 6\left( {1 + \frac{1}{\Lambda }} \right)}}{{dA_{1} }}} } \right)^{2} }} + \frac{{C_{2} }}{{\left( {\eta A_{2} + A_{1} \sqrt {\frac{{ - 6\left( {1 + \frac{1}{\Lambda }} \right)}}{{dA_{1} }}} } \right)^{3} }},$$

as $\eta \to 0$, $F\left( 0 \right) = 1$, Eq. (36) is simplified into another form as

37 $$F\left( \eta \right)\, = \,\frac{{C_{1} }}{{\left( {\eta A_{2} + A_{1} \sqrt {\frac{{ - 6\left( {1 + \frac{1}{\Lambda }} \right)}}{{A_{1} d}}} } \right)^{2} }} + \frac{{\left( {\frac{{ - 6\left( {1 + \frac{1}{\Lambda }} \right)}}{{dA_{1} }}} \right)^{\frac{3}{2}} - C_{1} \sqrt {\left( {\frac{{ - 6\left( {1 + \frac{1}{\Lambda }} \right)}}{{dA_{1} }}} \right)} }}{{\left( {\eta A_{2} + A_{1} \sqrt {\frac{{ - 6\left( {1 + \frac{1}{\Lambda }} \right)}}{{A_{1} d}}} } \right)^{3} }},$$

For a given value of $d$, there exists an enormous number of possible solutions for $F\left( \eta \right)$, and each one has an algebraically decaying function. Here $C_{1}$ is the independent variable, and the velocity components are then presented as

38 $$u\, = \,\frac{{\left( { - A_{1} 6\left( {1 + \frac{1}{\Lambda }} \right)} \right)}}{{A_{2} \left( {\eta A_{1} + A_{1} \sqrt {\frac{{ - 6\left( {1 + \frac{1}{\Lambda }} \right)}}{{A_{1} d}}} } \right)^{2} }}ax + C\left( {\,\frac{{C_{1} }}{{\left( {\eta A_{2} + A_{1} \sqrt {\frac{{ - 6\left( {1 + \frac{1}{\Lambda }} \right)}}{{A_{1} d}}} } \right)^{2} }} + \frac{{\left( {\frac{{ - 6\left( {1 + \frac{1}{\Lambda }} \right)}}{{dA_{1} }}} \right)^{\frac{3}{2}} - C_{1} \sqrt {\left( {\frac{{ - 6\left( {1 + \frac{1}{\Lambda }} \right)}}{{dA_{1} }}} \right)} }}{{\left( {\eta A_{2} + A_{1} \sqrt {\frac{{ - 6\left( {1 + \frac{1}{\Lambda }} \right)}}{{A_{1} d}}} } \right)^{3} }}} \right),$$

39 $$v\, = \, - \frac{{6A_{1} \left( {1 + \frac{1}{\Lambda }} \right)}}{{\eta A_{2} + A_{1} \left( {\sqrt {\frac{{ - 6\left( {1 + \frac{1}{\Lambda }} \right)}}{{A_{1} d}}} } \right)}}\sqrt {av} ,$$

For the algebraic expressions decaying condition, the non-dimensional stream function yieldscondition, the non-dimensional stream

40 $$\widetilde{\psi }\, = \,\frac{{6A_{1} \left( {1 + \frac{1}{\Lambda }} \right)x}}{{\eta A_{2} + A_{1} \sqrt {\frac{{ - 6\left( {1 + \frac{1}{\Lambda }} \right)}}{{A_{1} d}}} }} - \frac{C}{a}\left( {\,\frac{{C_{1} }}{{\left( {\eta A_{2} + A_{1} \sqrt {\frac{{ - 6\left( {1 + \frac{1}{\Lambda }} \right)}}{{A_{1} d}}} } \right)^{2} }} + \frac{{\left( {\frac{{ - 6\left( {1 + \frac{1}{\Lambda }} \right)}}{{dA_{1} }}} \right)^{\frac{3}{2}} - C_{1} \sqrt {\left( {\frac{{ - 6\left( {1 + \frac{1}{\Lambda }} \right)}}{{dA_{1} }}} \right)} }}{{\left( {\eta A_{2} + A_{1} \sqrt {\frac{{ - 6\left( {1 + \frac{1}{\Lambda }} \right)}}{{A_{1} d}}} } \right)^{3} }},} \right),$$

### Obtaining the temperature solution

Through integration, we resolve the temperature from Eq. (14) as

41 $$\theta \left( \eta \right)\, = \,1 - \frac{{\int_{0}^{\eta } {\exp \left( {\frac{{ - \Pr \beta tA_{4} }}{{A_{3} \left( {1 + N_{r} } \right)}}} \right) - \left( {\frac{{\Pr dA_{4} }}{{A_{3} \left( {1 + N_{r} } \right)\beta^{2} }}} \right)} }}{{\int_{0}^{\infty } {\exp \left( {\frac{{ - \Pr \beta tA_{4} }}{{A_{3} \left( {1 + N_{r} } \right)}}} \right) - \left( {\frac{{\Pr dA_{4} }}{{A_{3} \left( {1 + N_{r} } \right)\beta^{2} }}\exp \left( { - \beta \eta } \right)} \right)} }},$$

The relationship between thermal flux at the wall and the thermal efficiency at the wall reads

42 $$q_{w} \, = \, - k\left( {\frac{\partial T}{{\partial y}}} \right)_{y = 0} \, = \, - k\left( {T_{w} - T_{\infty } } \right)\sqrt{\frac{a}{v}} \theta ^{\prime}\left( 0 \right),$$

and after simplification we obtain

43 $$- \,\left( {\frac{\partial \theta }{{\partial \eta }}} \right)_{\eta = 0} = \,\frac{{\exp \left( {\frac{{ - \Pr dA_{4} }}{{\beta^{2} A_{3} \left( {1 + N_{r} } \right)}}} \right)}}{{\int_{0}^{\infty } {\exp \left( { - \frac{{\Pr \beta tA_{4} }}{{A_{3} \left( {1 + N_{r} } \right)}} - \left( {\frac{{ - \Pr dA_{4} }}{{\beta^{2} A_{3} \left( {1 + N_{r} } \right)}}} \right)\exp [ - \beta \eta ]} \right)} }},$$

As shown below, the definite integral in the denominator can be represented as,

44 $$\begin{aligned} & \int_{0}^{\infty } {\exp \left( {\left( {\frac{{ - \Pr \beta tA_{4} }}{{A_{3} \left( {1 + N_{r} } \right)}}} \right) + \left( {\frac{{\Pr \left( {V_{c} - \beta } \right)A_{4} }}{{A_{3} \left( {1 + N_{r} } \right)}}} \right)\exp [ - \beta \eta ]} \right)} \, \\ & \quad = \frac{{\left( {\frac{{\left( {\Pr dA_{4} } \right)}}{{\beta^{2} }}} \right)^{ - \Pr } }}{{A_{3} \left( {1 + N_{r} } \right)}}\left( {\Gamma \left( {\frac{{\Pr \beta tA_{4} }}{{A_{3} \left( {1 + N_{r} } \right)}},0} \right) - \Gamma \left( {\frac{{\Pr \beta tA_{4} }}{{A_{3} \left( {1 + N_{r} } \right)}},\frac{{\Pr \beta tA_{4} }}{{A_{3} \left( {1 + N_{r} } \right)\beta^{2} }}} \right)} \right) \\ \end{aligned}$$

where the incomplete gamma function is taken as $\Gamma \left( {a,x} \right)$. In this equation, it is critical to determine the integrals for the temperature profile. Using a variable transformation technique, a different method to the problem might be taken. A parameter $\xi = \Pr \left( {\frac{\exp [ - \beta \eta ]}{{\beta^{2} }}} \right),$ is exploited in order to resolve this problem. Thus, Eq. (14) becomes

45 $$\xi \frac{{d^{2} \theta }}{{d\xi^{2} }} + \left( {1 - \frac{{\Pr A_{4} }}{{A_{3} \left( {1 + N_{r} } \right)}} + \frac{{d\xi A_{4} }}{{A_{3} \left( {1 + N_{r} } \right)}}} \right)\frac{d\theta }{{d\xi }}\, = \,0,$$

and the boundary constraints being

46 $$\theta \left( {\frac{\Pr }{{\beta^{2} }}} \right)\, = \,1,\quad \theta \left( 0 \right)\, = \,0.$$

Now, the thermal solution yields

47 $$\theta \left( \eta \right)\, = \,\frac{{\Gamma \left( {\frac{{\Pr A_{4} }}{{A_{3} \left( {1 + N_{r} } \right)}},0} \right) - \Gamma \left( {\frac{{\Pr A_{4} }}{{A_{3} \left( {1 + N_{r} } \right)}},\frac{{dA_{4} \exp [ - \beta \eta ]}}{{A_{3} \left( {1 + N_{r} } \right)\beta^{2} }}} \right)}}{{\Gamma \left( {\frac{{\Pr A_{4} }}{{A_{3} \left( {1 + N_{r} } \right)}},0} \right) - \Gamma \left( {\frac{{d\Pr A_{4} }}{{A_{3} \left( {1 + N_{r} } \right)\beta^{2} }}} \right)}},$$

with respect to $\eta$, Eq. (47) differentiates as

48 $$\frac{d\theta }{{d\eta }}\, = \,\frac{{ - \beta \exp \left[ {\frac{{ - \Pr dA_{4} }}{{A_{3} \left( {1 + N_{r} } \right)}}} \right]\exp [ - \beta \eta ]\left( {\left( {\frac{{\Pr dA_{4} }}{{A_{3} \left( {1 + N_{r} } \right)\beta^{2} }}} \right)\exp [ - \beta \eta ]} \right)^{\Pr } }}{{\Gamma \left( {\frac{{\Pr A_{4} }}{{A_{3} \left( {1 + N_{r} } \right)}},0} \right) - \Gamma \left( {\frac{{\Pr A_{4} }}{{A_{3} \left( {1 + N_{r} } \right)}},\,\frac{{\Pr dA_{4} }}{{A_{3} \left( {1 + N_{r} } \right)\beta^{2} }}} \right)}},$$

Furthermore, the rate of heat transfer at the wall is given by

49 $$- \left( {\frac{\partial \theta }{{\partial \eta }}} \right)_{\eta \, = 0} = \,\frac{{\beta \exp \left( {\frac{{\Pr A_{4} }}{{A_{3} \left( {1 + N_{r} } \right)\beta^{2} }}} \right)\left( {\frac{{\Pr A_{4} d}}{{A_{3} \left( {1 + N_{r} } \right)\beta^{2} }}} \right)^{\Pr } }}{{\Gamma \left( {\frac{{\Pr A_{4} }}{{A_{3} \left( {1 + N_{r} } \right)\beta^{2} }},0} \right) - \Gamma \left( {\frac{{\Pr A_{4} }}{{A_{3} \left( {1 + N_{r} } \right)\beta^{2} }},\frac{{\Pr A_{4} d}}{{A_{3} \left( {1 + N_{r} } \right)\beta^{2} }}} \right)}},$$

Here, we observe that Eq. (49) is similar to Eq. (43), and this is further evidence of the reliability of the proposed method.

## Results and discussion

The precise solution extracted in this work provides a comprehensive explanation for phenomena occurring due to the stretched/shrinking surface. The surface velocity has been extended beyond purely linear conditions to more typical circumstances, with uniform wall transforming velocity. Both positive and negative values of *C* (the component of constant velocity) are possible to appear. As a result, the sheet motion may initiate by shrinking up to a specified distance from the slit and keep on changing either to stretching or shrinking. By employing the similarity transformation to the nonlinear PDEs, nonlinear ODEs are constructed. This transformation generates several physical variables, each of which is altered. Here $\varphi$ shows the presence and absence of the volume fraction of the fluid. A detailed description of the velocity and temperature distribution follows.

### Velocity profiles

**Figure 2:**
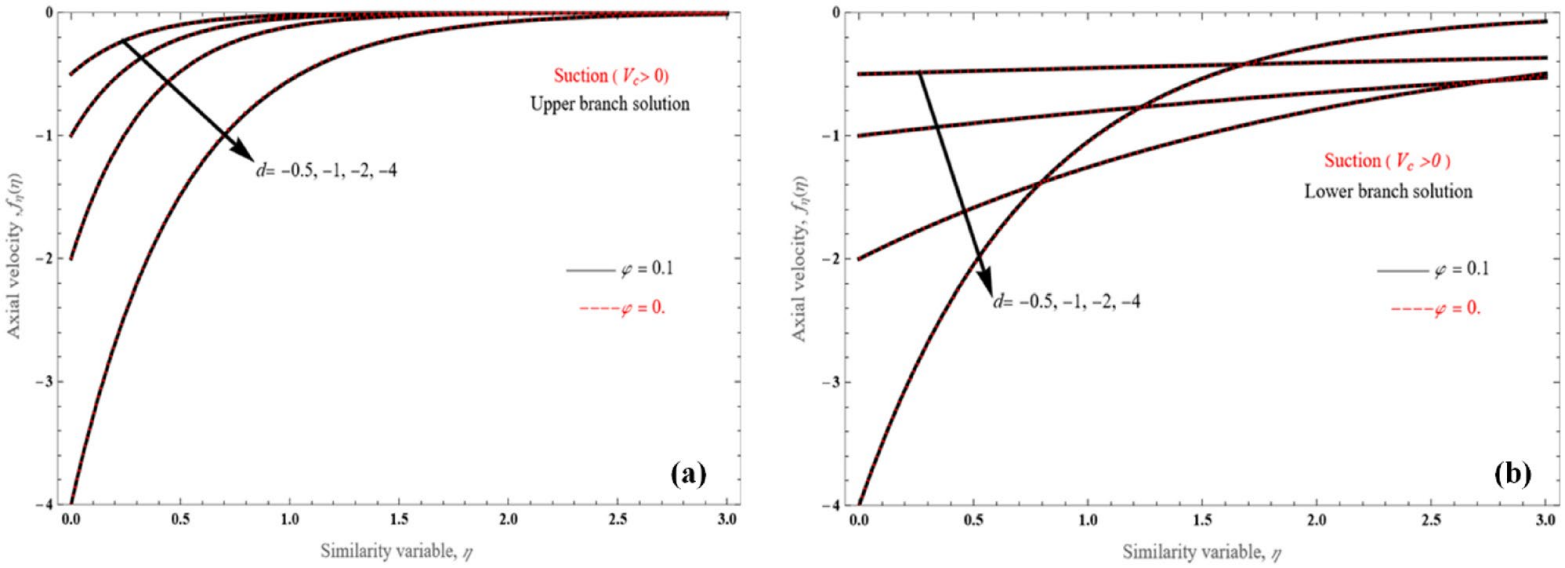
The tangential velocity patterns for the (**a**) upper branch and (**b**) lower branch solution, for various $d$ values.

It is shown that for the upper branch solution (Fig. 2a), alpha constant varies from − 0.5 to the single value for $V_{c} = 5$, which corresponds to $d = - 4$. Velocity profiles reveal that the fluid perforates more easily the surface for lower values of alpha. Nonetheless, its lower solution branch (Fig. 2b), differs significantly from the upper solution branch (Fig. 2a). Another point worth mentioning is that there are velocity profiles that do not follow a pattern as *d* decreases from − 0.5 to − 4, but cross to each other. Furthermore, for the same $V_{c}$ and stretching/shrinking parameter value, the upper branch solution has dramatically less momentum penetration than the lower branch solution.

**Figure 3:**
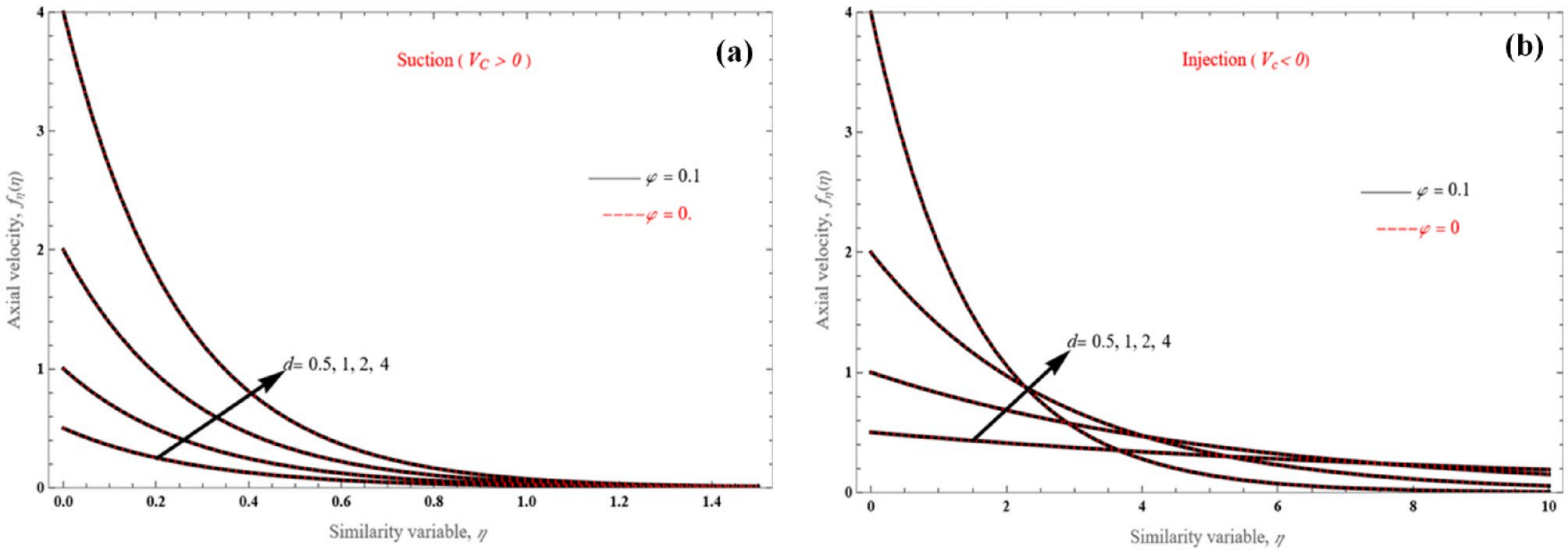
Axial velocity profiles for various values of $d$, for (**a**) $V_{c} > 0$ and (**b**) $V_{c} < 0$.

For the stretching layer ($d$ > 0) problem depicted in Fig. 3a,b, for both $V_{c} > 0$ and $V_{c} < 0$, when there is mass suction, velocity profiles reveal that fluid penetration is relatively small, and the stretching/shrinking parameter effect on the penetrated length are less apparent. However, according to Eq. (33), under specific $V_{c} > 0$ and for larger *d* values, the penetrated distance is reduced (Fig. 3a). In similar manner, for larger *d* values, the penetrating distance decreases for $V_{c} < 0$ (Fig. 3b), but there exist cross-sections in the velocity profiles.

**Figure 4:**
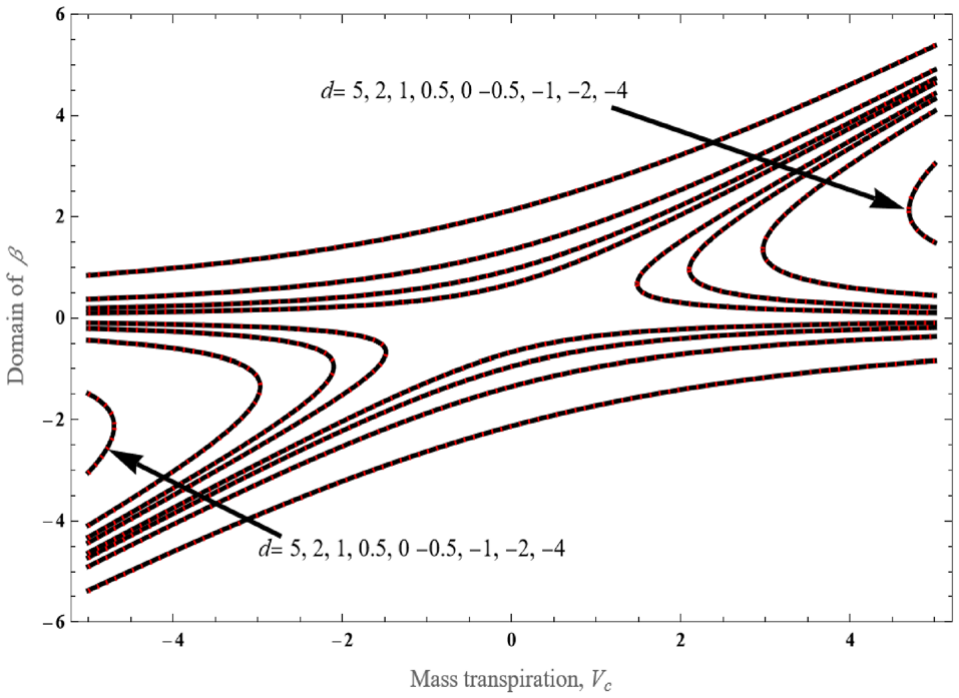
Solution region of $\beta$ vs. $V_{c}$ for various values of $d$.

Figure 4 presents the solution domain $\beta$ versus mass transpiration *V*_*c*_ for various *d* values. Here we observe that as the mass suction length increases, the upper branch result produces a higher value of $\beta$ for negative values of $d$. There is only a single solution for positive values of $d$, and this solution pertains to both mass injection and suction. The value of the domain of $\beta$ is altered inversely to the boundary layer thickness. Moreover, the upper branch $\beta$ solution decreases and falls in a negative value of $d$ for a particular value of $V_{c} > 0$, denoting greater rate of sheet contraction.

**Figure 5:**
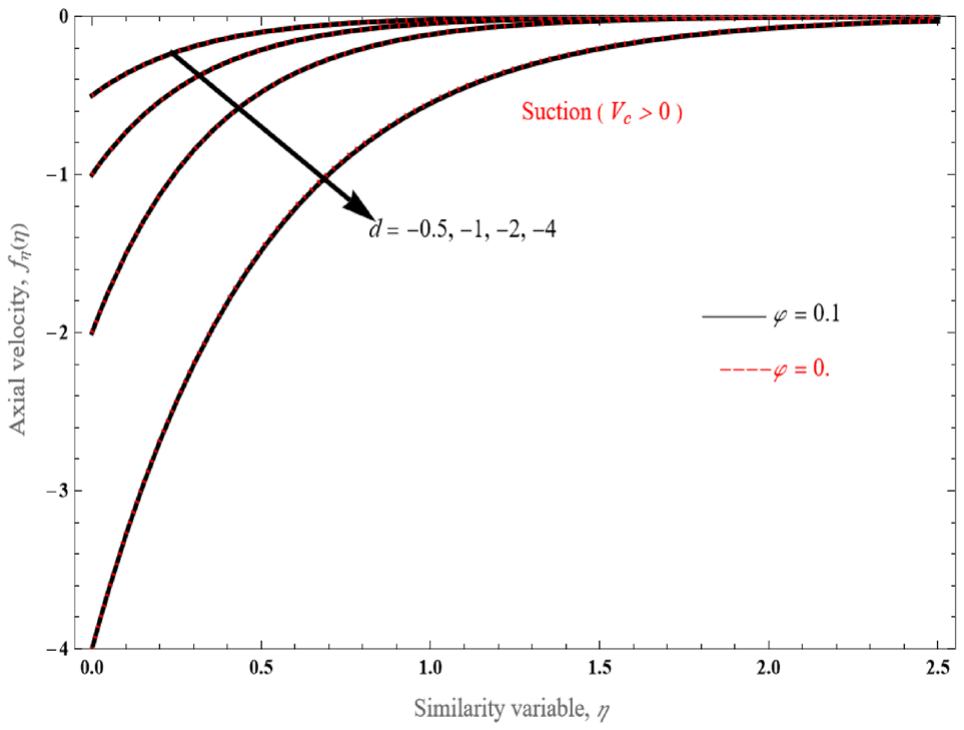
The axial velocity profiles for the algebraically decaying solutions for various values of $d$.

In comparison to the exponential decaying solution shown in Fig. 4, the algebraically decaying solution, $F\left( \eta \right)$, in Fig. 5 is considerably different.

It can be seen that the velocity profiles exhibit rather substantial flow penetration to the surrounding fluid. Interesting findings are made for function *F*'s flow due to the wall's constant velocity component. Based on Eq. (36), when $C_{1}$, the solution reduces to very simple form:

50 $$F\left( \eta \right)\, = \frac{{\left( {\frac{{ - 6\left( {1 + \frac{1}{\Lambda }} \right)}}{{dA_{1} }}} \right)^{\frac{3}{2}} }}{{\left( {\eta A_{2} + A_{1} \sqrt {\frac{{ - 6\left( {1 + \frac{1}{\Lambda }} \right)}}{{A_{1} d}}} } \right)^{3} }},$$

**Figure 6:**
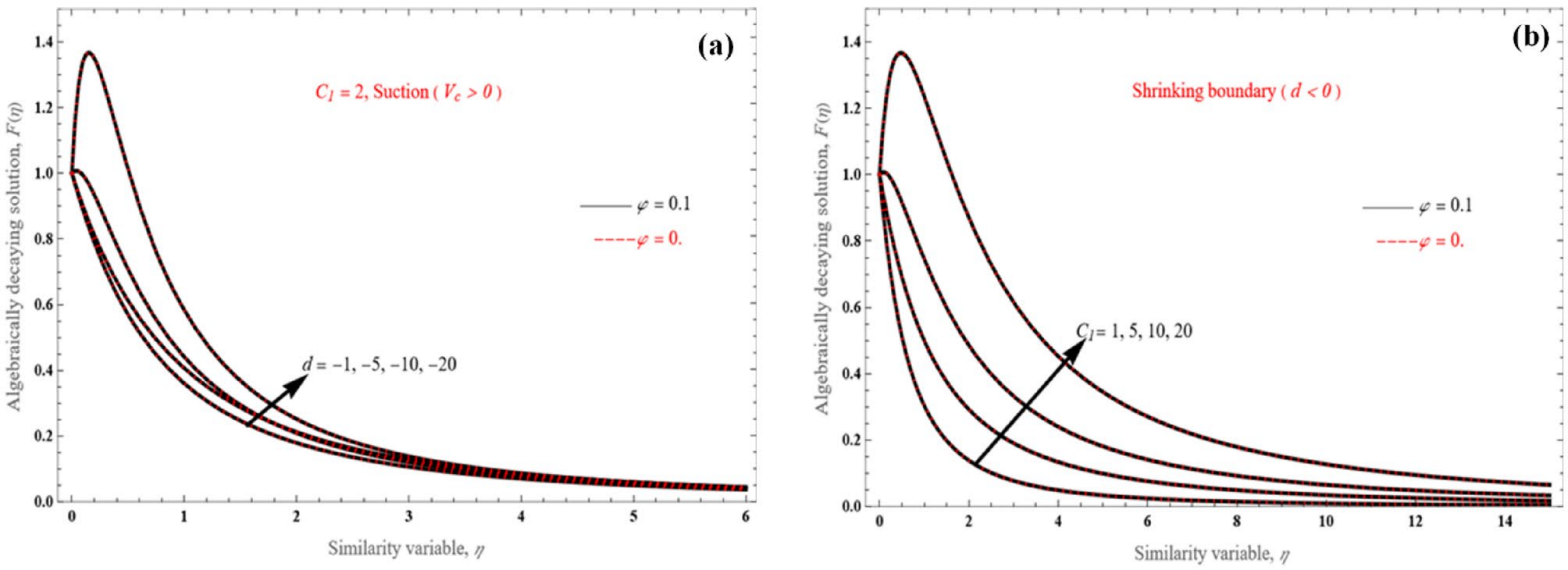
$F(\eta )$ vs. the similarity variable for (**a**) various values of $d$ and (**b**) various positive values of $C_{1}$.

Positive values of $C_{1}$ and $d$ have an impact on the velocity distribution, as shown in Fig. 6. The steep velocity increase close to the wall is an interesting result. This phenomenon takes place for higher positive values of $C_{1}$ and $d$.

**Figure 7:**
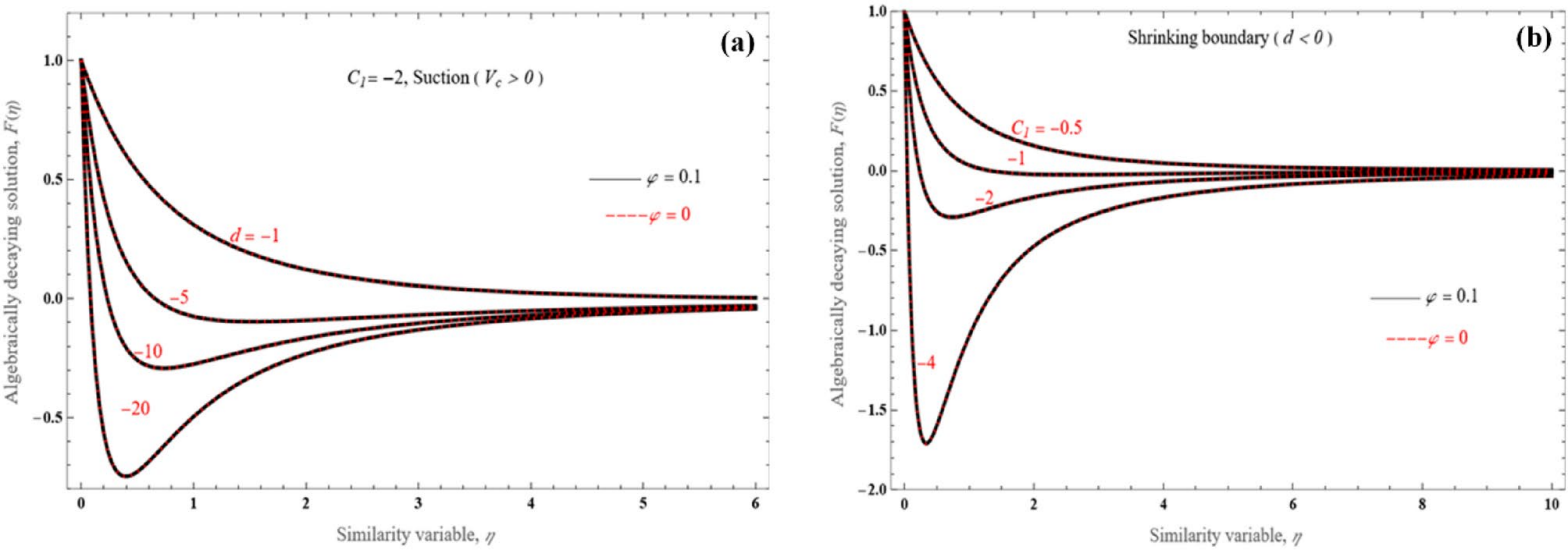
$F(\eta )$ vs. the similarity variable for (**a**) various values of $d$ and (**b**) various negative values of $C_{1}$.

On the other hand, when $C_{1}$ is negative (Fig. 7a,b), there is analogous steep velocity decrease towards the negative direction, with reverse flow in the boundary layer. This is a novel outcome, which has not been observed before for the algebraically decaying solution. In order to analyze the variation characteristics of the profile, the derivative of algebraically decaying solution can be obtained as follows

51 $$\frac{\partial F}{{\partial \eta }}\, = \,\frac{{ - 2C_{1} }}{{\left( {\eta A_{2} + A_{1} \sqrt {\frac{{ - 6\left( {1 + \frac{1}{\Lambda }} \right)}}{{A_{1} d}}} } \right)^{3} }} - 3\frac{{\left( {\frac{{ - 6\left( {1 + \frac{1}{\Lambda }} \right)}}{{dA_{1} }}} \right)^{\frac{3}{2}} - C_{1} \sqrt {\left( {\frac{{ - 6\left( {1 + \frac{1}{\Lambda }} \right)}}{{dA_{1} }}} \right)} }}{{\left( {\eta A_{2} + A_{1} \sqrt {\frac{{ - 6\left( {1 + \frac{1}{\Lambda }} \right)}}{{A_{1} d}}} } \right)^{4} }},$$

Therefore, it is revealed that a negative velocity overshoot for a negative value of $C_{1}$ might develop.

To conclude on velocity profiles presented in this Section, we have demonstrated that velocity values are significantly affected by various physical parameters in the modeled system, such as the stretching/shrinking parameter and the suction/injection parameter.

### Temperature profiles

**Figure 8:**
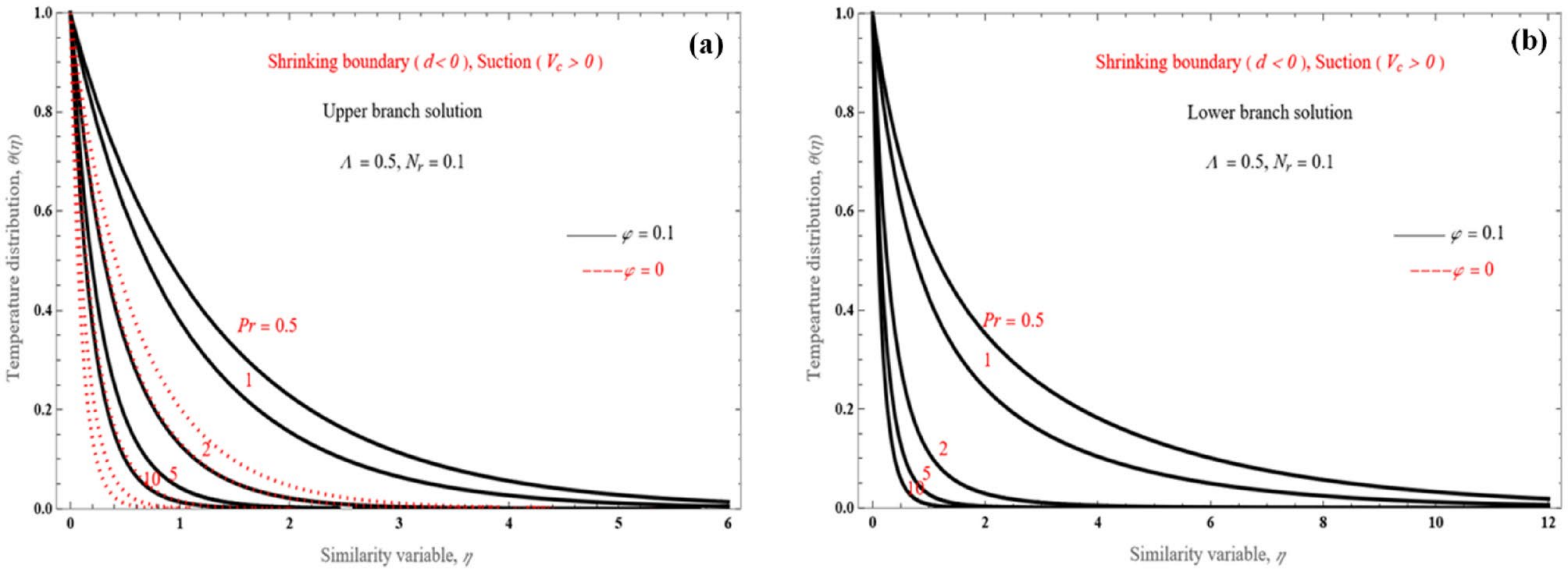
Temperature distribution for the (**a**) upper branch solution and (**b**) lower branch solution, for the shrinking sheet case and various value of Pr.

The effect of the Prandtl number on temperature distribution is shown in Fig. 8a,b. Here we depict the non-dimensional temperature distribution for both the two solution branches for the shrinking layer design with $d = - 2$ and $V_{c} = 4$, for various values of Pr. More specifically, the thermal boundary layer thickness decreases as Pr increases for both solutions. The lower branch solution (Fig. 8b) has a marginally broader thermal boundary layer compared to the upper branch solution (Fig. 8a).

**Figure 9:**
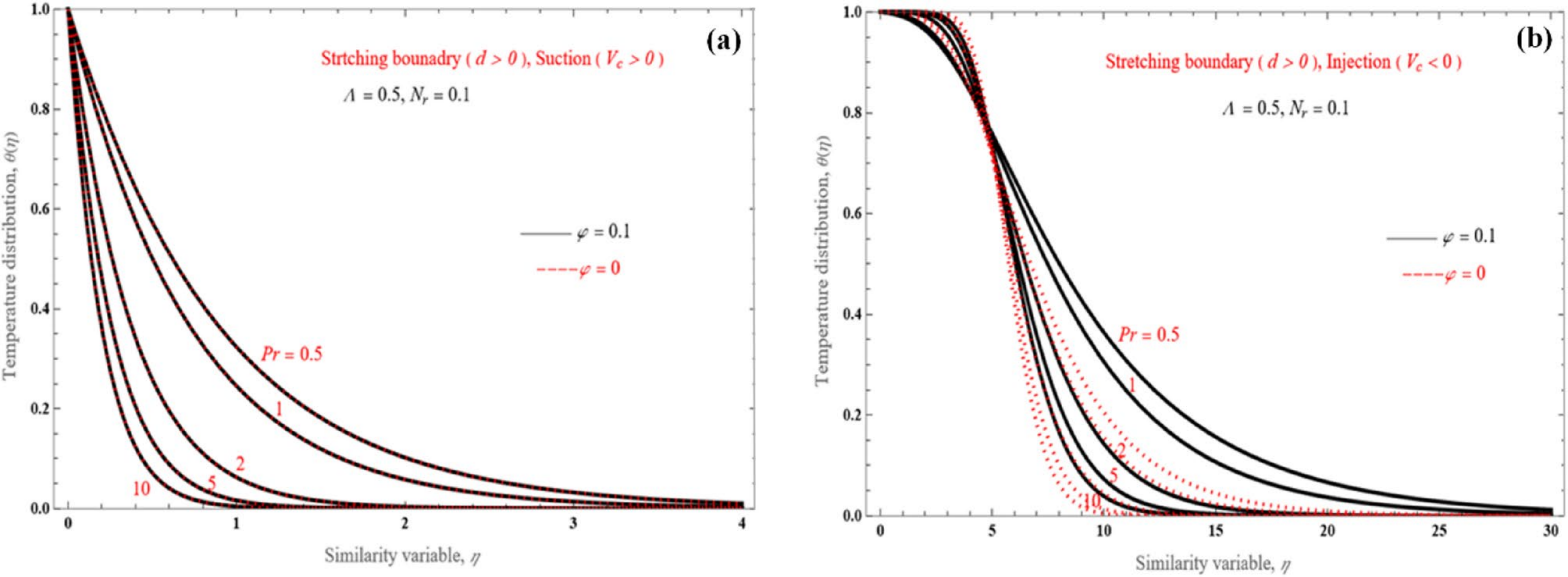
Temperature distribution for the (**a**) upper branch solution with suction and (**b**) lower branch solution with injection, for the shrinking sheet problem and various Pr values.

Comparisons between suction ($V_{c} > 0$) and injection ($V_{c} < 0$) cases for the stretching boundary problem are presented in Fig. 9a,b. The wall boundary layer is blasted away by mass injection (Fig. 9b), and this fact results in significantly low heat flow at the surface. Even when the Pr gets higher values, the boundary layer thickness still remains small. Another point worth mentioning is the shape of the temperature profile as Pr increases; the wall heat transfer rate decreases because of a thinner heated wall, and the temperature falls steeply to ambient temperature.

**Figure 10:**
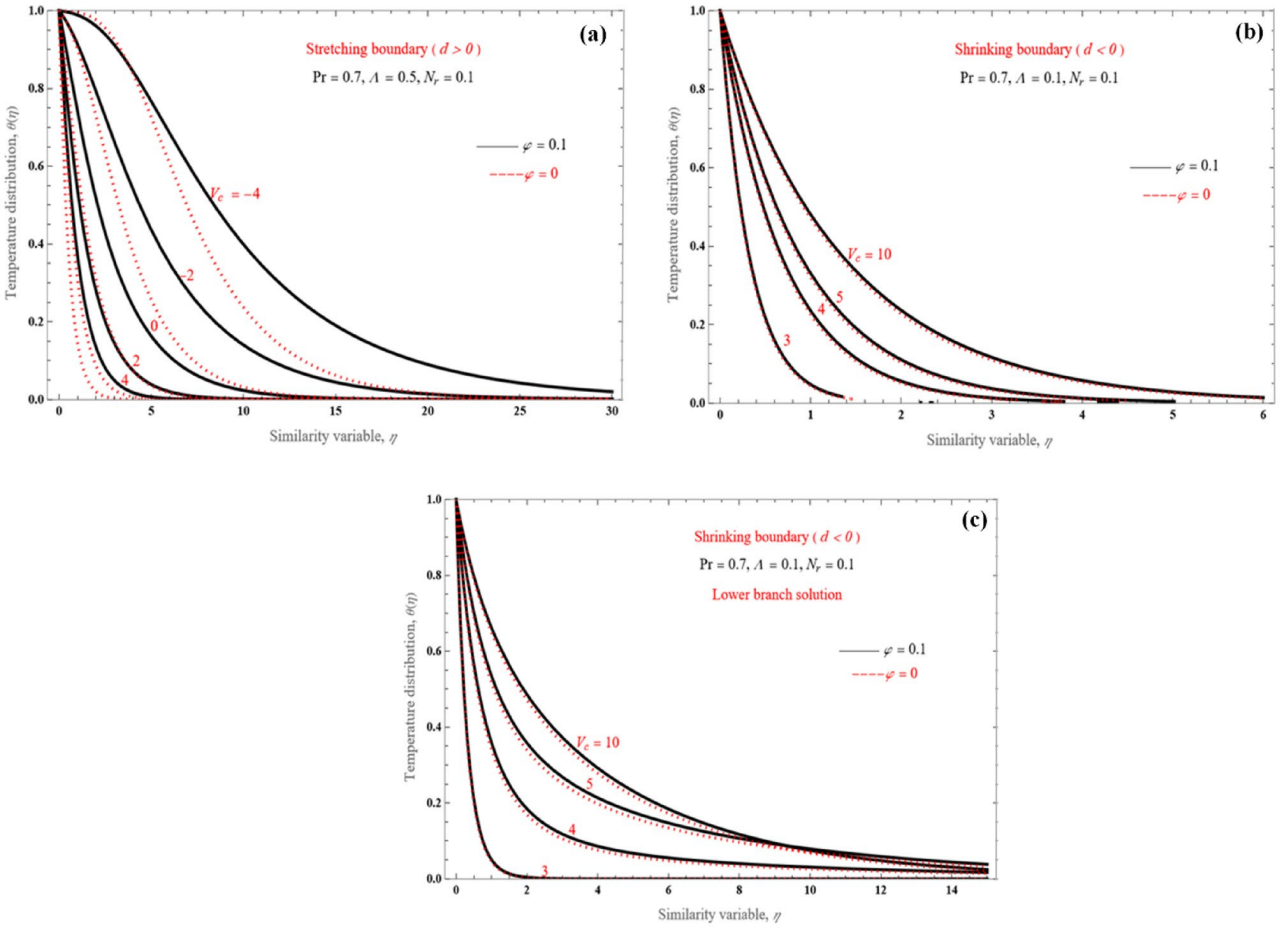
The effect of $V_{c}$ on temperature profile for (**a**) upper branch solution and $d > 0$, (**b**) upper branch solution and $d < 0$, (**c**) lower branch solution and $d < 0$.

We further investigate the effect of the mass transfer variable value on the temperature profiles in Fig. 10a–c. Figure 10a presents both mass injection and suction cases as *V*_*c*_ changes from -4 to 4, for sheet stretching. The wall heat flux decreases as *V*_*c*_ increases, for Pr = 0.7, Λ = 0.5, and *N*_*r*_ = 0.1. As the fluid reaches the boundary layer, we observe an increase in its thickness. We can obtain solutions only for Eq. (31), that is $V_{c} \ge \sqrt { - 6A_{1} d\left( {1 + \frac{1}{\Lambda }} \right)}$ for a diminishing surface problem, unfortunately. Heat flow at the wall and the boundary layer thickness are comparable for both solution branches. In Fig. 10a–c, it is observed that the boundary layer gradually decreases for both branches and temperature values fall rapidly as mass suction increases. Although this is not observed for the upper branch solution, for the lower branch solution there are some overlaps between temperature profiles, in Fig. 10c.

**Figure 11:**
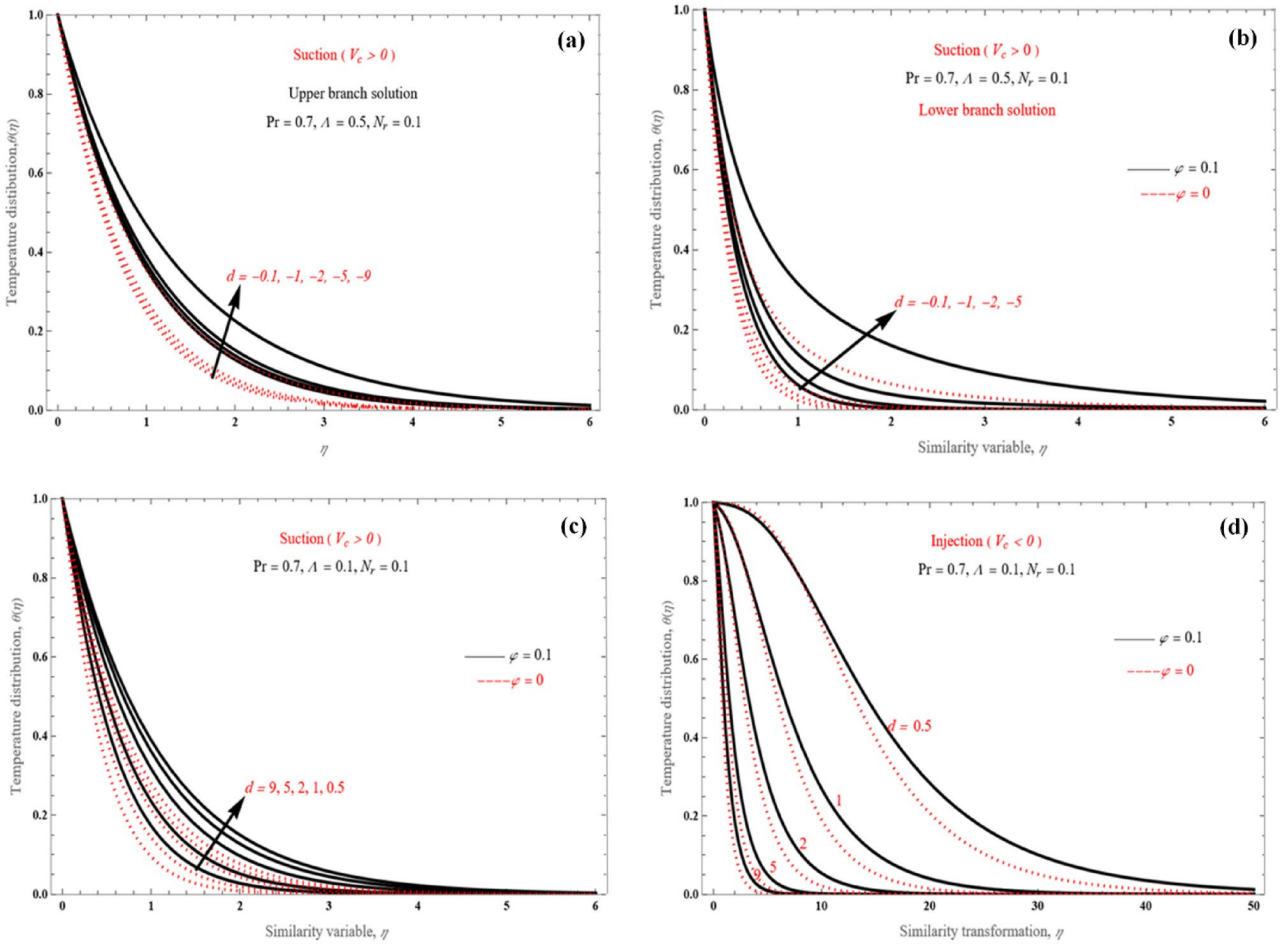
Temperature profiles for (**a**) shrinking sheet, upper branch solution, (**b**) shrinking sheet, lower branch solution, (**c**) stretching sheet, suction, and (**d**) stretching sheet, injection case.

Figure 11a–d show how the wall strength can expand or contract. Both the upper (Fig. 11a) and lower solutions (Fig. 11b) present similar behavior for the shrinking problem. Confirmed cross-over positions across the few different temperature patterns are for the lower solution branches. For the stretching sheet, the behavior is different for suction (Fig. 11c, $V_{c} > 0$) and injection (Fig. 11d, $V_{c} < 0$). With an increase in stretched strength at the wall and a corresponding rise in surface heat transfer rate, the boundary layers for mass injection and suction become both smaller, while wall heat flux increases.

**Figure 12:**
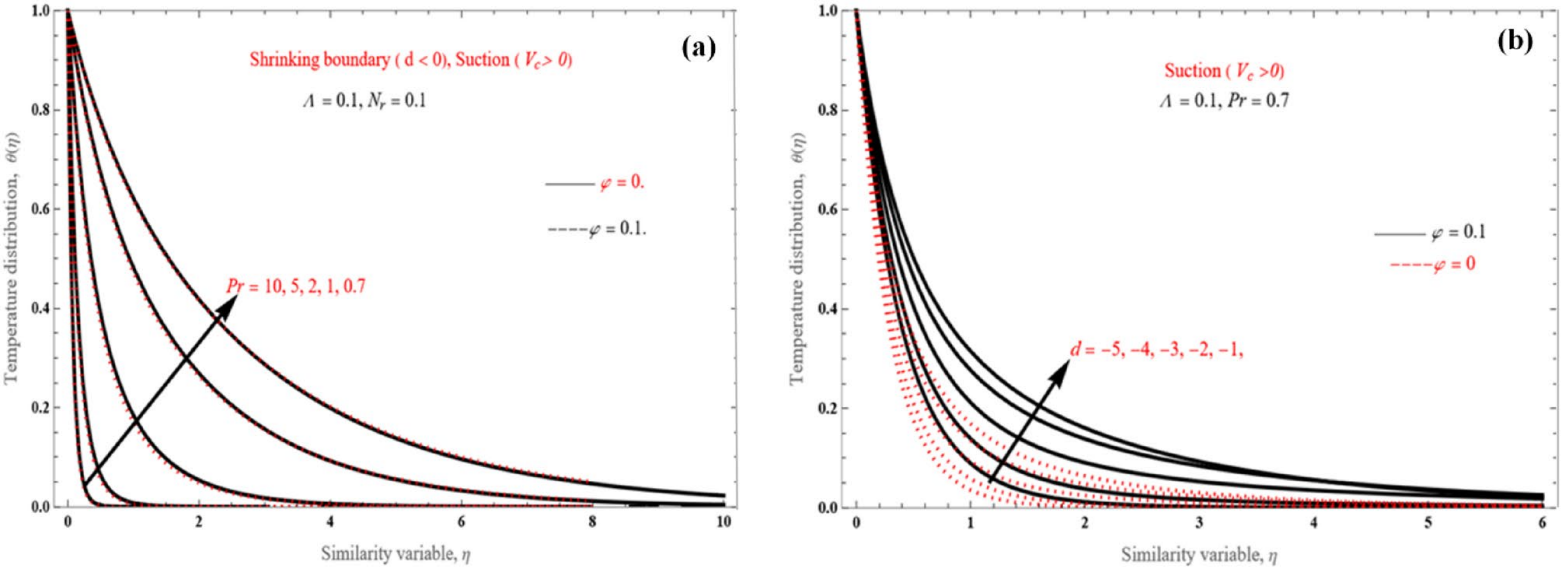
Temperature distribution (**a**) for various Pr values in shrinking case and (**b**) for various *d* values.

Figure 12a,b illustrate some instances of the algebraically declining temperature field. Under a specific decreasing strength, the boundary layer thickness decreases for higher values of Pr (Fig. 12a). Moreover, it is observed that the boundary layers become wider as *d* increases from − 5 to − 1.

**Figure 13:**
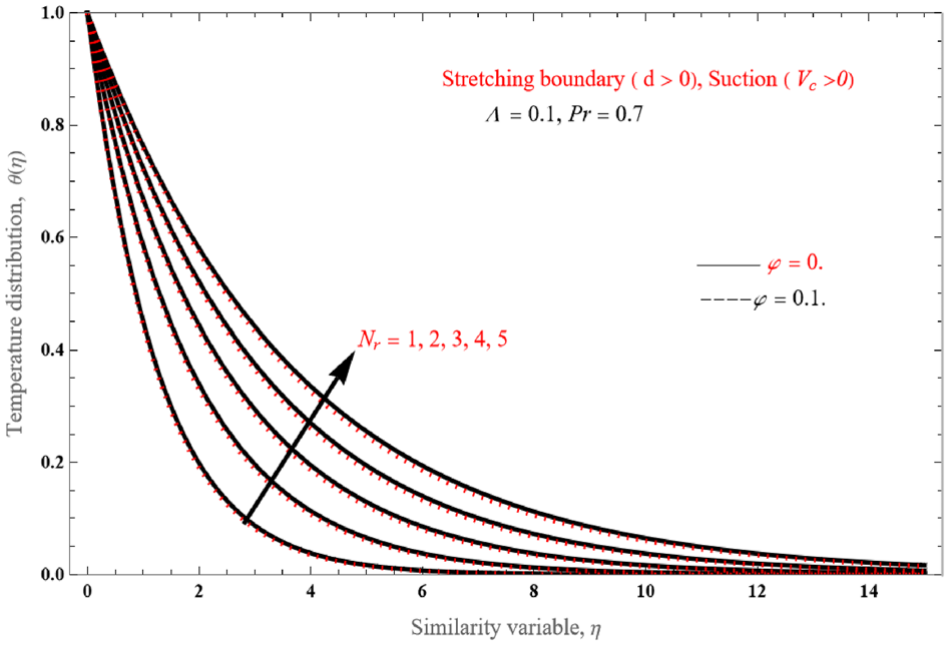
Temperature profiles for various values of the radiation parameter $N_{r}$.

The impact of temperature on the radiation parameter $N_{r}$ is shown in Fig. 13, where temperature values increase as $N_{r}$ increases from 1 to 5. This is attributed to the fact that an increase in the radiation parameter allows for more heat transfer through the fluid.

Our investigation has shown that when there is a high fluid flow rate, fluid particles tend to collide with each other, decreasing the boundary layer thickness when the temperature increases.

### Flow streamlines

**Figure 14:**
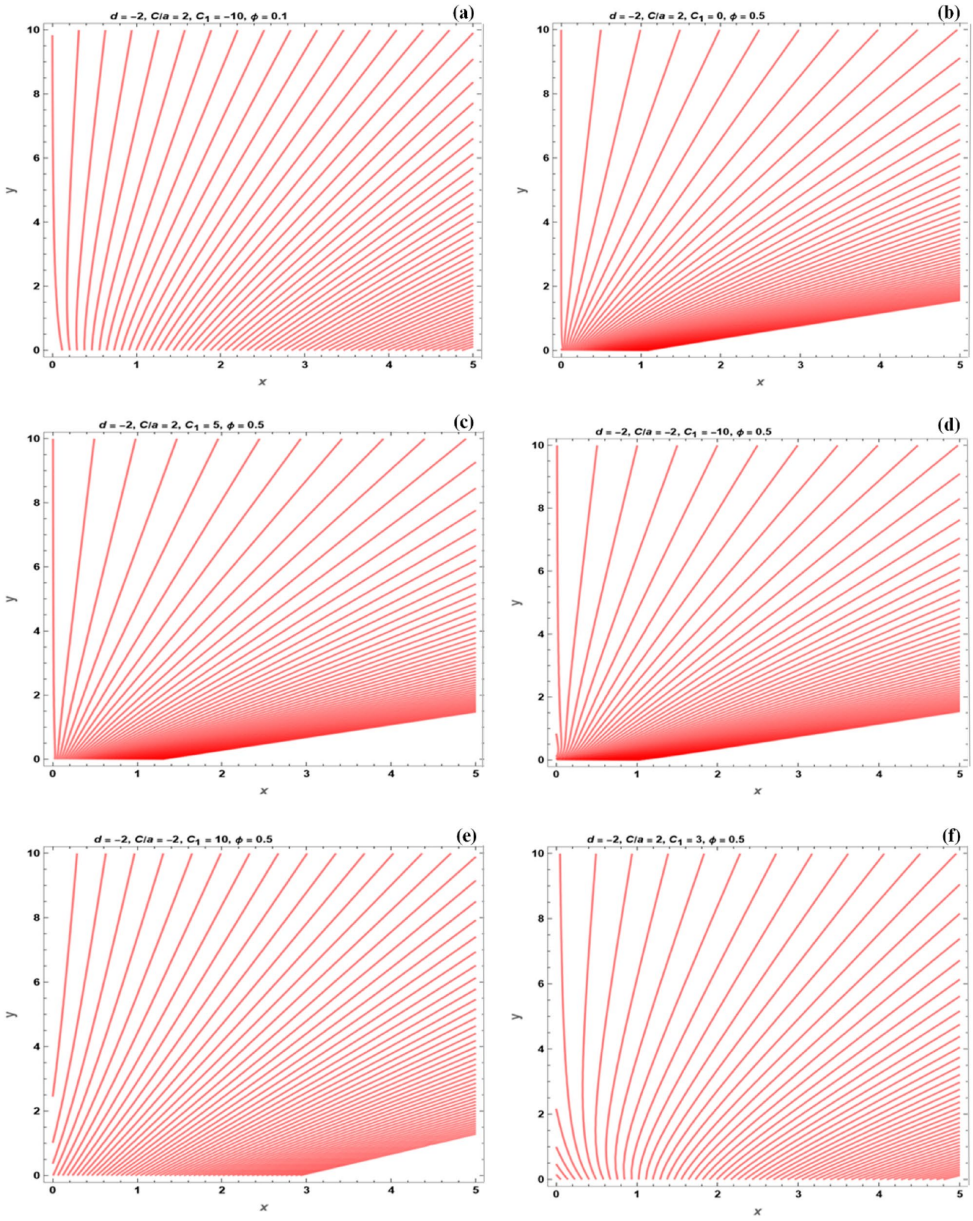
The flow field under the effect of various parameters, for the shrinking sheet.

**Figure 15:**
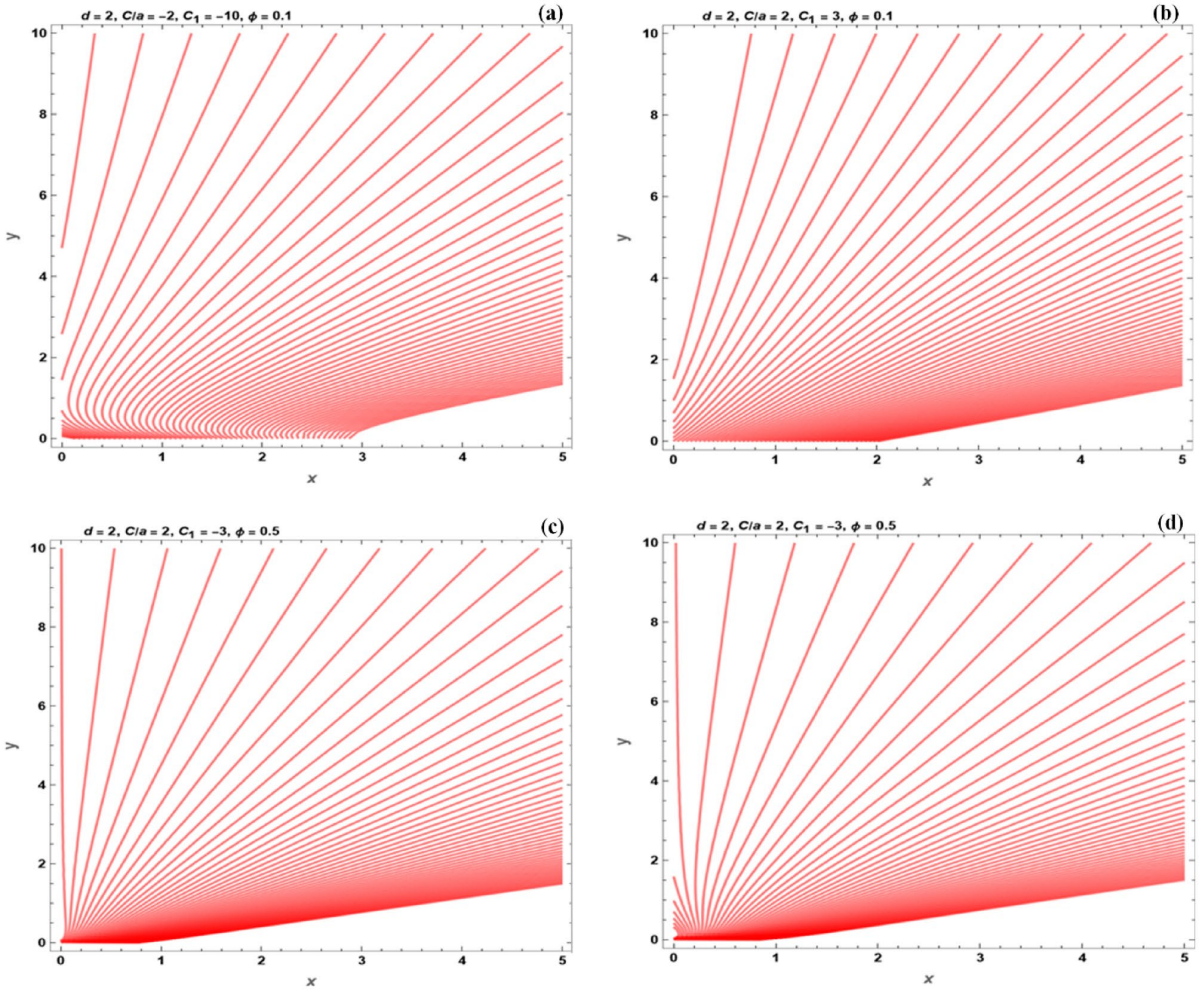
The flow field under the effect of various parameters, for the stretching sheet.

Figures 14 and 15 present the flow field of the dimensionless stream functions for various values of the stretching/shrinking factors, $\frac{C}{a}$. For the shrinking problem (Fig. 14), it is observed that when the fluid is expanded towards the edge of both solution branches, the $\frac{C}{a}$ value is negative. The sheet moves away from the slot for positive values of $\frac{C}{a}$, before moving back towards the slot after a particular distance. There is a point with $u = 0$ on the sheet for both solution branches at $x = \frac{C}{b}$.

On the other hand, the stretching surface problem is depicted in Fig. 15a–d. Flow patterns for various mass injection/suction are depicted. There is a point for $u = 0$, with a combination of a positive *a* and negative $\frac{C}{a}$ under mass suction. The fluid is initially stretched in the direction of the slot, then after it has passed that point, it is extended outward from the slot. When $\frac{C}{a}$ is positive and negative, the fluid is always extended away from the slot. At a particular distance from the wall, the vertical velocity becomes zero, when mass injection is applied.

The flow field analysis can enhance our understanding on how a fluid moves over a stretching/shrinking sheet. And this is a significant step towards increasing productivity, operational effectiveness and work conditions, by removing irrelevant or ineffective processes.

## Conclusions

The objective of this paper has been the investigation on the effect of radiation on the flow of viscous Casson nanofluids over a linear stretching/shrinking surface, with mass transfer parameter. Aluminum oxide is considered as the nanoparticle additive in the flow model. The governing partial differential equations have been transformed into ordinary differential equations via similarity transformations, and accurate analytical solutions have been provided, to promote our understanding on the physical processes hidden behind these expressions.

Current findings have revealed numerous solutions for the flow field. Exponential and algebraically decaying solutions have been illustrated. We have presented a number of solution branches for flow field as well as velocity overshoots. Velocity and temperature profiles, along with a flow field representation, are extracted in various conditions of the problem, such as for suction/injection cases, shrinking/stretching sheet strength, various values for the Prandtl number, and the radiation parameter value, and they are found to be significantly affected by each one of these parameters. Highlighted results of the present research work are listed as follows:

- Stretching/shrinking parameter value decreases with decreasing the momentum of the boundary layer in both cases of upper and lower solution branch, while the algebraically solution is also decreasing.
- Stretching/shrinking parameter value increases when the momentum of the boundary layer increases in both cases of suction and injection, but the exactly opposite behavior occurs in temperature profiles.
- The effect of Prandtl number, Pr, is found to reduce the thickness of the temperature boundary layer.
- As a result of thermal radiation, the temperature profile values increase rapidly.
- For a stretching problem, improved in mass transpiration, the thermal penetration becomes thicker. For upper and lower branches, the thermal boundary layer thickness decreases when mass transpiration increases.

To capture the contribution of the present work, we have extended the field of application as:

- $$\mathop {\lim }_{{\Lambda \to \infty ,q_{r} \to 0}} \left\{ {Results of present work} \right\} \to$$ {*Results of Fang *et al*.*^17,30–32^}.
- On replacing the nanofluid by the hybrid nanofluid in the absence of Casson parameter, we get the result of Mahabaleshwar et al.^29^.

## Data Availability

Data that support the findings of this study are available from the corresponding author upon reasonable request.

## Latin symbols

*a*: Constant
*C*: Component of constant velocity
$C_{p}$: Specific heat (J K^−^^1^ kg^−^^1^)
$$C_{1}$$: Free parameter (–)
$d$: Stretching/shrinking parameter
$$f$$: Similarity function
$F$: Algebraically decaying variable
$k^{*}$: Mean absorption coefficient (cm^−^^1^)
$$N_{r}$$: Radiation factor
Pr: Prandtl number
$q_{r}$: Radiative heat flux (W m^−^^2^)
$$T$$: Fluid temperature (K)
$T_{w}$: Temperature of the surface (K)
$$T_{\infty }$$: Ambient temperature (K)
$u$: Component of velocity along x-axis (m s^−^^1^)
$v$: Component of velocity along y-axis (m s^−^^1^)
$V_{c}$: Mass transfer variable
$$x$$: Horizontal axis
$$y$$: Vertical axis
$\Lambda$: Casson fluid parameter
$\left( {\rho C_{p} } \right)$: Heat capacitance of fluid (J kg^−^^1^ K)

## Greek symbols

$$\eta$$: Similarity variable
$$\mu$$: Dynamic viscosity (N s m^−^^2^)
$$\nu$$: Kinematic viscosity (m^2^ s^−^^1^)
$\rho$: Fluid density (kg m^−^^3^)
$$\varphi$$: Volume fraction (–)
$$\theta$$: Fluctuating temperature (K)
$$\psi$$: Stream function (K)
$\sigma^{*}$: Stefan–Boltzmann constant (W m^−^^2^ K^−^^4^)

## Subscripts/superscripts

$$f$$: Base fluid
$$nf$$: Nano fluid
$$u_{r}$$: Reference velocity

## Code availability

Codes that support the findings of this study are available from the corresponding author upon reasonable request.
